# A CD8^+^ T cell related immune score predicts survival and refines the risk assessment in acute myeloid leukemia

**DOI:** 10.3389/fimmu.2024.1408109

**Published:** 2024-09-12

**Authors:** Zeyi Li, Peng Jin, Rufang Xiang, Xiaoyang Li, Jie Shen, Mengke He, Xiaxin Liu, Hongming Zhu, Shishuang Wu, Fangyi Dong, Huijin Zhao, Han Liu, Zhen Jin, Junmin Li

**Affiliations:** ^1^ Shanghai Institute of Hematology, State Key Laboratory of Medical Genomics, National Research Center for Translational Medicine at Shanghai, Ruijin Hospital Affiliated to Shanghai Jiao Tong University School of Medicine, Shanghai, China; ^2^ Department of General Practice, Ruijin Hospital Affiliated to Shanghai Jiao Tong University School of Medicine, Shanghai, China; ^3^ Wuxi Branch of Ruijin Hospital Affiliated to Shanghai Jiao Tong University School of Medicine, Shanghai, China

**Keywords:** acute myeloid leukemia, CD8^+^ T cell, the European LeukemiaNet, prognosis, CIBERSORTx

## Abstract

Although advancements in genomic and epigenetic research have deepened our understanding of acute myeloid leukemia (AML), only one-third of patients can achieve durable remission. Growing evidence suggests that the immune microenvironment in bone marrow influences prognosis and survival in AML. There is a specific association between CD8^+^ T cells and the prognosis of AML patients. To develop a CD8^+^ T cell-related immune risk score for AML, we first evaluated the accuracy of CIBERSORTx in predicting the abundance of CD8^+^ T cells in bulk RNA-seq and found it significantly correlated with observed single-cell RNA sequencing data and the proportions of CD8^+^ T cells derived from flow cytometry. Next, we constructed the CTCG15, a 15-gene prognostic signature, using univariate and LASSO regression on the differentially expressed genes between CD8^+^ T^High^ and CD8^+^ T^Low^ groups. The CTCG15 was further validated across six datasets in different platforms. The CTCG15 has been shown to be independent of established prognostic markers, and can distill transcriptomic consequences of several genetic abnormalities closely related to prognosis in AML patients. Finally, integrating this model into the 2022 European LeukemiaNet contributed to a higher predictive power for prognosis prediction. Collectively, our study demonstrates that CD8^+^ T cell-related signature could improve the comprehensive risk stratification and prognosis prediction in AML.

## Introduction

Acute myeloid leukemia (AML) is a heterogeneous hematopoietic malignancy with diverse genetic abnormalities (1). New standard therapies such as Azacitidine and Venetoclax have enhanced survival for older, unfit AML patients at initial diagnosis. Despite these improvements, most AML patients eventually relapse, with survival rates of 32% at two years and 24% at five years (2). Novel therapeutic strategies, such as checkpoint blockade (3) and chimeric antigen receptor T-cell therapies (4), have achieved promising impacts on the outcomes in hematologic malignancies. Similarly, numerous ongoing clinical trials are exploring the effectiveness of stimulating the immune system in the treatment of AML (5–7).

To date, our understanding of the classification of AML has been based on the somatic mutations and cytogenetic abnormalities, which is also the cornerstone of the European LeukemiaNet (ELN) genetic risk classification (8, 9). Nevertheless, leukemia is not just a genetic disorder; it represents a complex microenvironment within the bone marrow, consisting of tumor cells, various immune cells, and other cell types (10). Recently, studies have addressed the significance of immune microenvironment in AML, disclosing that the immune-related genes may predict the therapeutic response and patients’ prognosis (11–13). T cells in the bone marrow (BM) are the prerequisite for anti-leukemia response. It has been reported that the percentage and the dysfunctional state of T cells in BM were correlated with the response to treatment and the survival rates (14–17). Given the substantial influence of CD8^+^ T cells on both the efficiency of immunotherapy and prognosis in AML, we intend to explore whether a prognostic model built on CD8 T-related genes can accurately forecast the survival rates of AML patients.

In order to accurately evaluate CD8^+^ T cells in AML BM, we chose CIBERSORTx as the machine learning method that enables the estimation of cell type abundances from bulk transcriptome data (18, 19). CIBERSORTx, a computational framework for digitally isolating individual cell type transcriptomes from mixed bulk RNA data without physical separation, now includes functions for cross-platform data normalization and virtual cell purification (18). CIBERSORTx, widely employed as an effective tool in AML research for quantifying immune cell abundance (20–22), has been confirmed for its accuracy compared to other methods (21, 22). Utilizing this method, we calculated CD8^+^ T cell abundance in AML BM specimens.

In our study, we aim to build a CD8 tumor infiltrating lymphocytes (TILs) related prognostic score which is strongly correlated with overall survival (OS) in AML patients. Firstly, we used four distinct methods to validate the accuracy of CIBERSORTx’s prediction of CD8 TILs abundance from bulk RNA-seq. We then analyzed RNA-sequencing data from AML patients in the RJAML cohort (RNA-sequencing data of bone marrow samples from *de novo* AML patients collected at Ruijin Hospital) and generated fifteen CD8 TILs-related genes from the differentially expressed genes (DEGs) to construct the CTCG15 prognostic model. We demonstrated the effectiveness of this model across seven distinct cohorts from RJAML, HOVON, TCGA-LAML, BeatAML, and GEO. Further, we explored the relationship between CTCG15 and the high-frequency mutations in AML. Finally, we integrated CTCG15 into the ELN2022 framework, this resulted in a notable enhancement in the predictive accuracy of ELN. Through our findings, we aim to provide CD8 TILs insights that could refine risk stratification in AML.

## Methods

### Analysis of scRNA-seq

Before conducting single-cell data analysis, we extracted the immune cells types and expression counts based on the annotations provided in the original text (23). The single-cell RNA sequencing (scRNA-seq) data were normalized using the ‘Seurat’ R package (24), followed by log-transformation with an offset of 1 and subsequent scaling. We identified genes with substantial expression variation by employing the ‘FindVariableFeatures’ function in Seurat, specifying ‘vst’ as the value for the ‘vst.method’ parameter. Then, we utilized ‘ScaleData’ function to normalize the gene expression values. We performed linear dimensionality reduction on the high-dimensional dataset through Principal Component Analysis (PCA) on the highly variable genes. The ‘IntegrateLayers’ was applied to mitigate batch effects across different experimental batches. We then applied the Uniform Manifold Approximation and Projection (UMAP) algorithm for nonlinear dimensionality reduction and visualized the distribution of the data in the reduced-dimensional space. Utilizing ‘FindMarkers’, we identified genes with statistically significant differences in expression across various groups.

### Collection and RNA-sequencing of AML patient samples

We collected bone marrow samples from 157 patients newly diagnosed with AML at Ruijin Hospital, Shanghai, China, during the period from June 2019 to September 2020 (**Supplementary Table S6**). The collection of the specimens was approved by the Institutional Review Boards of Ruijin Hospital, and the written informed consent for specimen collection and research was obtained following the Declaration of Helsinki (RJ-AML2014-65 & RJ-AML2016). RNA was extracted by AllPrep PowerFecal RNA Kit, and RNA-seq libraries were prepared with TruSeq RNA Sample Preparation Kit v2 (Illumina, San Diego, CA, USA). The quality of the RNA was assessed using an RNA 6000 Bioanalyzer Nano Kit (Agilent, Palo Alto, CA, USA). Sequencing was conducted on Illumina’s NovaSeq 6000, with adapter-trimmed reads aligned using STAR (v2.7.8) and quantified with HT-Seq (v0.13.5) against GRCh38. Expression levels were normalized to TPM with a custom script. In this manuscript, the cohort is designated as ‘RJAML.’ The details of the examined loci are available for review in **Supplementary Table S7**.

### Patient cohorts

The scRNA-seq data of AML BM cells from 16 patients were acquired from GSE116256 (23) along with the bulk RNA-seq data of these 16 patients. The HOVON cohort [n = 618; ref. (25)] was obtained from Array Express (Dataset ID: E-MTAB-3444) and used as the training set. Gene expression profiles and survival information were extracted from the GSE146173 [n = 246; ref. (26)], GSE37642 [n = 553; ref. (27)], GSE12417 [n = 240; ref. (28)] cohorts from GEO. TCGA-LAML [n = 179; ref. (29)] and BeatAML [n = 244; ref. (30)] cohorts were accessed through the GDC data portal for the RNA-sequencing (RNA-seq) data, clinical information, and processed mutational variants. It is noteworthy that, only samples from newly diagnosed adult AML patients were retained in all cohorts, and patients with incomplete survival information in these cohorts were omitted.

### Estimation of immune cell proportion

The CIBERSORTx (18) algorithm was employed to calculate the relative abundance scores for non-leukemic immune cell types based on the bulk RNA-seq data of AML BM. Specifically, gene expression data from these cells was processed with CIBERSORTx to create a signature matrix, with the minimum expression parameter being tuned to 0.25 from the default settings. Deconvolution was applied to TPM-normalized RNA-seq data using S-mode batch correction in absolute mode and the relative abundance scores were subsequently normalized to indicate the proportion of each cell type.

### Flow cytometry analysis

Twenty-four BM specimens from AML patients were randomly selected from the aforementioned 157 RJAML samples. Specimens were thawed and then washed with Dulbecco’s phosphate-buffered saline (DPBS) containing 2% fetal bovine serum. Cells were stained with multiple monoclonal antibodies including anti-human CD45-PE, CD3-BV510, and CD8-APC-CY7 at 4°C for 30 minutes. Before FCM analysis, samples were further washed to remove antibodies resuspended in staining buffer. In the flow cytometry analysis, a minimum of 10,000 cells were collected for each sample.

### Differential gene expression analysis and enrichment analysis

Differential expression analysis, comparing CD8^+^ T^High^ and CD8^+^ T^Low^ patients, were conducted using the raw count data with the DESeq2 R package. We identified DEGs using a cutoff of |logFC|>1 and FDR<0.05, finding 2925 DEGs. GO (Gene Ontology) and KEGG (Kyoto Encyclopedia of Genes and Genomes) analyses of these DEGs were conducted using ‘ClusterProfiler’. Enrichment pathways from both GO and KEGG with p-values and q-values below 0.05 were considered significant.

### Construction of a prognostic signature based on CD8^+^ T cells related genes

The normalized gene expression profiles of CD8^+^ T cells from AML patients in the HOVON cohort were used to serve as the training set. Univariate cox analysis of OS was performed to screen for CD8^+^ T cells genes with potential prognostic value. Significant candidate genes were refined using the least absolute shrinkage and selection operator (LASSO) model, which was implemented by the glmnet package, to identify a subset of pivotal genes for constructing a predictive model. The LASSO model selected 15 key genes and their regression coefficients were utilized to calculate the CTCG15 score for each sample. Patients were stratified into high-risk and low-risk groups based on the median threshold derived from the CTCG15 scores.

### Statistical analyses

Statistical analyses were performed by R software (version 4.0.3). The Wilcoxon rank-sum test assessed differences between two groups. The package “survival” and “survminer” were used to determine the significance of survival analysis. We utilized Kaplan-Meier plots and log-rank tests to evaluate the impact of the signature on OS and event-free survival (EFS). The univariate regression was utilized to select genes of prognostic value with the “survival” package. A two-tailed P-value < 0.05 indicated statistical significance.

### Data availability statement

The original data collected in Ruijin Hospital is accessible in the GEO database under accession number GSE201492.

## Results

### Validation of CIBERSORTx’s ability of immune cell deconvolution by comparing with scRNA-seq results

**Figure 1:**
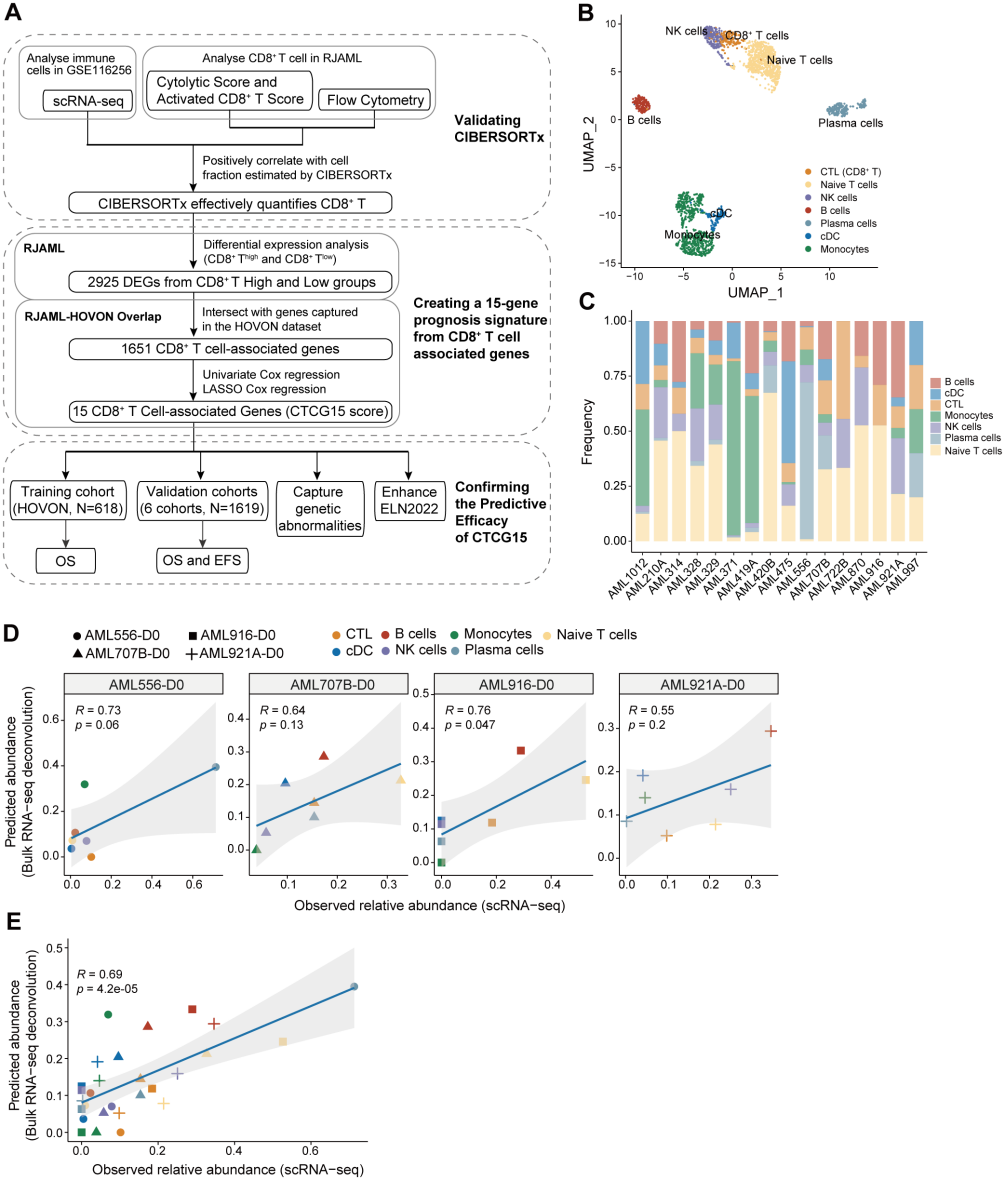
Validating the accuracy of CIBERSORTx to deconvolute the immune cells in acute myeloid leukemia (AML) bulk RNA-seq. **(A)** A flowchart for study design. **(B)** Uniform Manifold Approximation and Projection (UMAP) clustering map of bone marrow (BM) immune cells from 16 AML patients in GSE116256 shows the distribution of 7 cellular clusters, representing 7 types of immune cells. **(C)** Bar plot displaying the frequencies of seven types of immune cells from **(B)** separately in 16 AML patients in GSE116256. **(D)** Pearson correlation between observed abundance from scRNA-seq and predicted abundance from bulk RNA-seq for each of the four patients separately in GSE116256. The bulk RNA-seq profile utilized patient-specific reference signatures derived from the scRNA-seq data of each respective patient. **(E)** The Pearson correlation was performed between observed and predicted abundance for four patients combined from **(D)**.

For better understanding, the workflow of this study was illustrated in **Figure 1A**. Firstly, our goal was to verify the accuracy of CIBERSORTx in measuring CD8^+^ T cell levels in RNA-seq data. We found that CIBERSORTx’s results correlate positively with scRNA-seq data, two RNA-seq scoring methods (Cytolytic Score and Activated CD8^+^ T Score), and flow cytometry. Secondly, we developed a prognostic model with 15 CD8^+^ T cell-related genes (CTCG15). After identifying 2925 DEGs in CD8^+^ T cell expression groups, we used the HOVON cohort to refine these genes. The CTCG15 score was then created using univariate and Lasso Cox regression analyses. Finally, we validated the predictive power of CTCG15 score across multiple cohorts, confirming its ability to forecast patient survival and identify prognostic genetic markers. It also improves the predictive accuracy of the ELN2022 scoring system.

We analyzed the scRNA-seq data from GSE116256 (23) and classified the immune cells into 7 types: CD8^+^ T cells, natural killer (NK) cells, naive T cells, B cells, plasma cells, monocytes, and conventional dendritic cells (cDCs) (**Figure 1B**). The proportion of immune cells for each AML patient was calculated and shown in **Figure 1C**. We then employed CIBERSORTx (18) to deconvolute the bulk RNA-seq data of four patients in GSE116256 using matched patients-specific reference signatures derived from scRNA-seq data (31). For each patient, we performed Pearson correlation analysis between the predicted abundances from bulk RNA-seq data with CIBERSORTx and the observed abundances from scRNA-seq. Positive correlations were found between CIBERSORTx and scRNA-seq results in both individual (**Figure 1D**) and combined data of four AML patients (**Figure 1E**), which demonstrated the reliable performance of CIBERSORTx to deconvolute immune cells.

### Further validation of CIBERSORTx’s accuracy for CD8^+^ T cell analysis via additional algorithms and flow cytometry results

**Figure 2:**
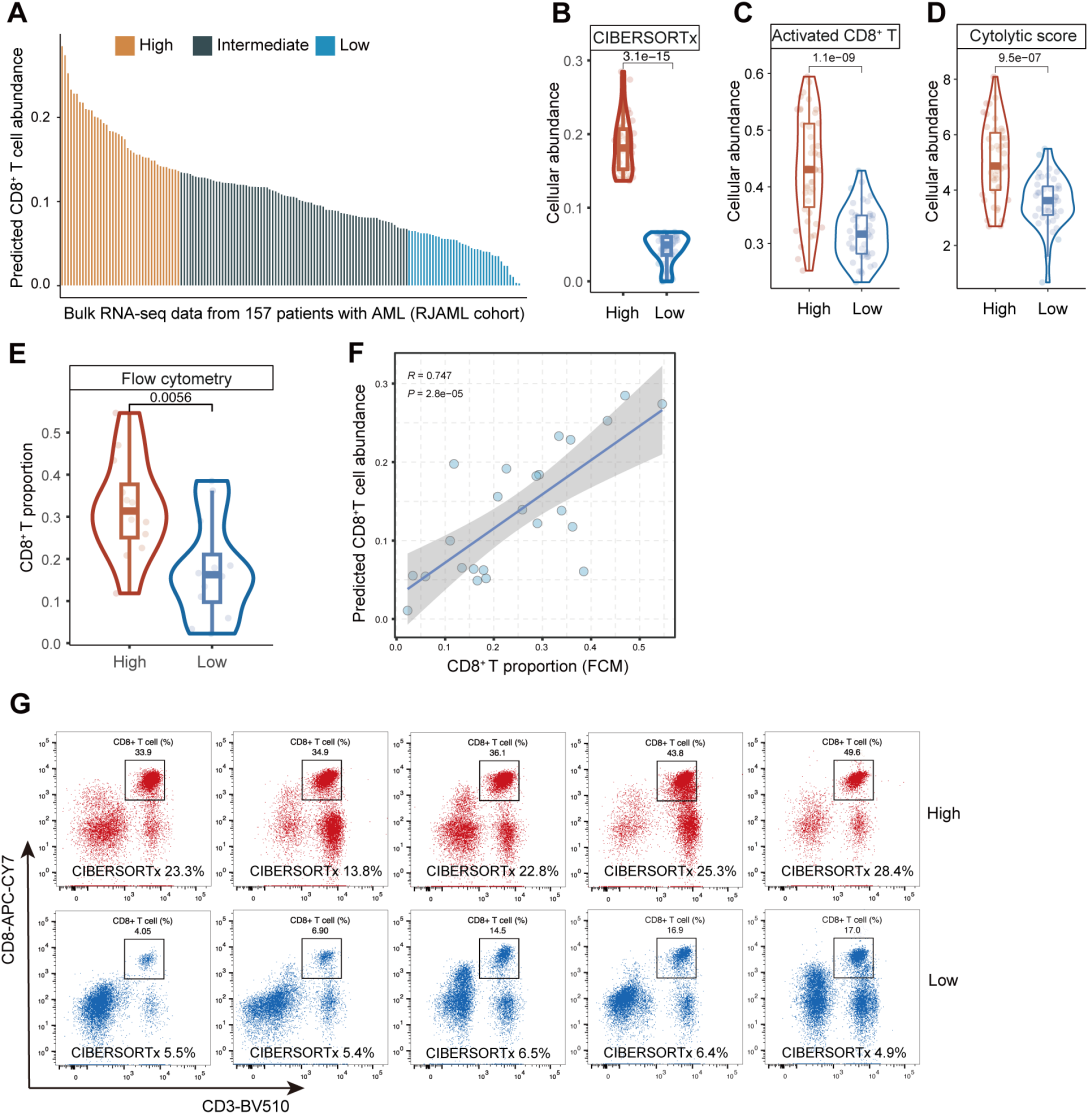
Assessment of predictive performance of CIBERSORTx in CD8 tumor infiltrating lymphocytes (TILs) from bulk RNA-seq data in RJAML cohort. **(A)** The predicted abundance of CD8^+^ T cells in RJAML with CIBERSORTx, and the three groups (High, Intermediate and Low) categorized based on quartiles. **(B–D)** The abundance of CD8^+^ T cells between CD8^+^ T^High^ and CD8^+^ T^Low^ groups calculated by CIBERSORTx, the Activated CD8^+^ T Score and the Cytolytic Score. **(E)** The comparison of CD8^+^ T cell proportions detected by flow cytometry (FCM) of 24 patients randomly selected from CD8^+^ T^High^ and CD8^+^ T^Low^ groups in RJAML (12 from the CD8^+^ T^High^ group and 12 from CD8^+^ T^Low^ group). **(F)** Pearson correlation between the CD8^+^ T proportion from CIBERSORTx and FCM of the 24 patients. **(G)** Ten representative CD8^+^ T cells FCM plots are shown in CD8^+^ T^High^ and CD8^+^ T^Low^ groups.

We employed other methods at both transcriptomic and proteomic levels to validate CIBERSORTx. To begin with, the predicted abundance of CD8^+^ T cells from the bulk RNA-seq data in the RJAML cohort was analyzed and quantified by the CIBERSORTx (18) (**Figure 2A**). It was stratified into three categories based on quartiles for subsequent analysis: High, Intermediate, and Low. In the analysis results from CIBERSORTx, the abundance of CD8^+^ T cells in the High group was significantly greater than that in the Low group (**Figure 2B**). This observation was further validated by two independent methods: the Activated CD8^+^ T Score (31) (**Figure 2C**) and the Cytolytic Score (32) (**Figure 2D**), which consistently demonstrated a significantly higher proportion of CD8^+^ T cells in the High group compared to the Low group. Next, twelve RJAML specimens were randomly selected from both the CTCG^High^ group and the CTCG^Low^ group for flow cytometry analysis, aiming to calculate the percentages of CD8^+^ T cells among non-leukemic immune cells. The FCM results indicate that the proportion of CD8^+^ T cells in the CD8^+^ T High group defined by CIBERSORTx is significantly higher than that in the Low group (**Figure 2E**), which is consistent with the results of CIBERSORTx in **Figure 2B**. Pearson correlation revealed a significant positive correlation between the predicted CD8^+^ T cell abundance from bulk RNA-seq deconvolution and the CD8^+^ T cell percentages from flow cytometry (**Figure 2F**). **Figure 2G** displays five representative flow cytometry plots for each group, and their tendencies align with the results obtained from CIBERSORTx. Overall, the capability of CIBERSORTx in evaluating CD8^+^ TILs has been further validated with the methods above.

### Development of a 15-gene prognostic signature derived from CD8^+^ T cell related genes in AML

**Figure 3:**
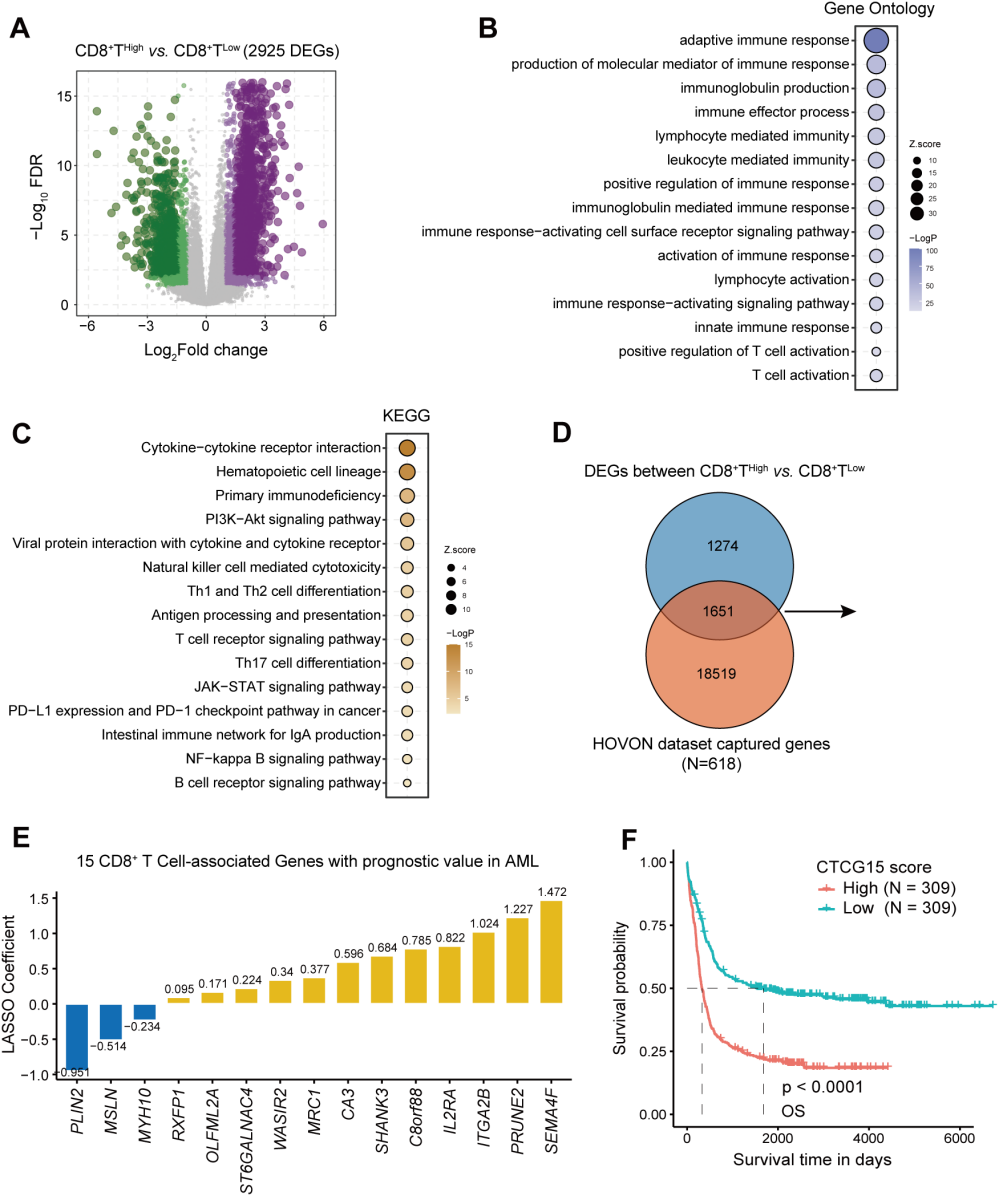
Identification of CD8^+^ T cell-related genes in AML and construction of 15-gene prognostic signature. **(A)** Volcano plot showing differentially expressed genes (DEGs) between groups with high and low CD8^+^ T cell levels, determined using the threshold of |log2(fold change)| ≥ 1 and FDR < 0.05. **(B, C)** Bubble chart displaying GO and KEGG analysis results for CD8^+^ T cell-related genes. **(D)** A Venn diagram identifying the overlap between DEGs and HOVON dataset. **(E)** Distribution of LASSO coefficients for prognosis-related genes. **(F)** Kaplan-Meier curves depicting the difference of overall survival (OS) between CTCG15^High^ and CTCG15^Low^ groups in the HOVON cohort.

To identify genes associated with CD8^+^ T cell, we performed differential analysis between the CD8 TILs High and Low groups with the DESeq2 package (32) and found a total of 2925 DEGs (with |logFC|>1 and FDR<0.05) (**Supplementary Table S1**, **Figure 3A**). We performed GO and KEGG enrichment analysis (**Supplementary Tables S2, S3**) to further elucidate the biological functions and to identify signaling pathways linked to these DEGs. The top 15 GO (**Figure 3B**) and KEGG (**Figure 3C**) terms associated with the DEGs cover various immune responses and immune cell activation pathways, which underline that DEGs are associated with immune reactions.

1651 CD8^+^ T cell genes were retained after we intersected 2925 DEGs with genes in the training cohort HOVON (25) (**Figure 3D**). Using univariate cox regression and LASSO regression, we yielded an optimal 15-gene CD8^+^ T cell signature (the CTCG15 score) as shown in **Figure 3E**. These fifteen genes involved are *PLIN2*, *MSLN*, *MYH10*, *RXFP1*, *OLFML2A*, *ST6GALNAC4*, *WASIR2*, *MRC1*, *CA3*, *SHANK3*, *C8orf88*, *IL2RA*, *ITGA2B*, *PRUNE2* and *SEMA4F*. Kaplan–Meier analysis revealed a significant association between high CTCG15 risk scores and reduced OS in the training cohort HOVON, suggesting its ability to effectively predict prognosis for AML patients (**Figure 3F**).

### Extensive assessment of the predictive ability of CTCG15 in multiple external cohorts

**Figure 4:**
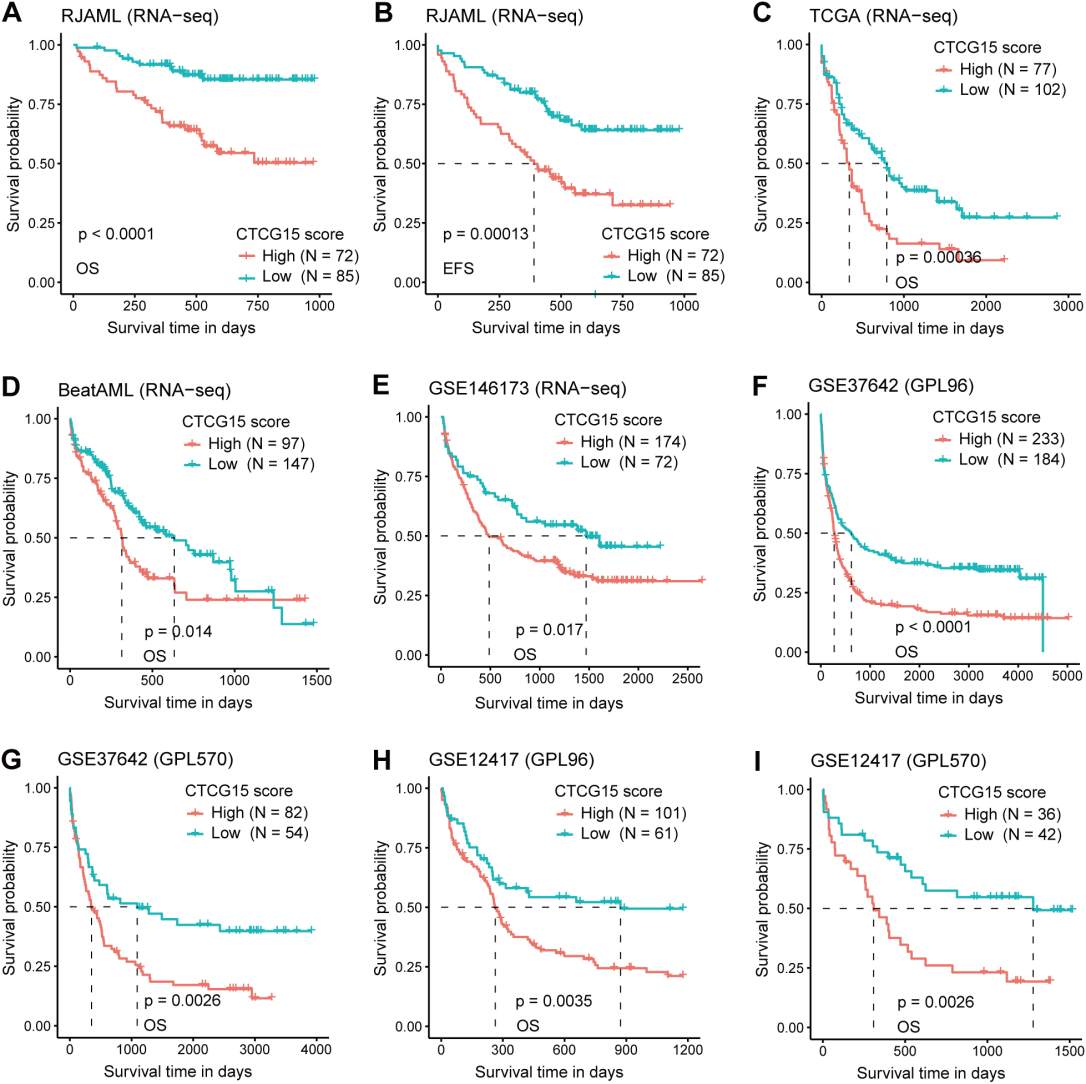
The CTCG15 score is strongly linked with OS and event free survival (EFS) across various independent AML cohorts in different analysis platforms. Kaplan–Meier curves depicting the difference of OS **(A)** and EFS **(B)** between CTCG15^High^ and CTCG15^Low^ groups in the RJAML cohort. Kaplan-Meier estimates of OS according to the CTCG15 score in the RNA-seq–based cohorts: TCGA **(C)**, BeatAML **(D)**, and GSE146173 **(E)**. Kaplan–Meier estimates of OS based on the CTCG15 score in the GSE37642 **(F, G)** and GSE12417 **(H, I)** cohorts, quantified on GPL96 and GPL570 microarray platforms.

After the development of CTCG15, we explored its correlation with OS and EFS in 1619 AML patients across six cohorts and two technology platforms. We first validated the CTCG15 score with the RNA-seq data of 157 *de novo* AML patients from the RJAML cohort. Consistent with our observations from the HOVON dataset, patients exhibiting high CTCG15 scores (CTCG15^High^) showed notably worse OS (**Figure 4A**) and EFS (**Figure 4B**) than those with low CTCG15 scores (CTCG15^Low^). When applied to other RNA-seq datasets including TCGA (n = 179) (29), BeatAML (n = 244) (30), and GSE146173 (n = 246) (26), the CTCG15 all remained strongly associated with clinical outcomes (**Figures 4C–E**). Similarly, the significant difference of OS between CTCG15^High^ and CTCG15^Low^ groups was also observed in the GSE37642 (n = 553) (27) (**Figures 4F, G**) and GSE12417 (n = 240) (28) (**Figures 4H, I**), with both datasets being analyzed using the GPL96 and GPL570 platforms. In multivariate survival analysis with Cox proportional hazards (CPH) models, the CTCG15 score demonstrated ability to constitute a novel independent prognostic indicator in the TCGA, BeatAML, and RJAML cohorts, apart from the established outcome markers like patient age, white blood cell (WBC) count, ELN2022, and the presence of *NPM1* and *FLT3-ITD* mutations (**Supplementary Table S4**).

### CTCG15 captured specific genetic abnormalities related to AML prognosis

**Figure 5:**
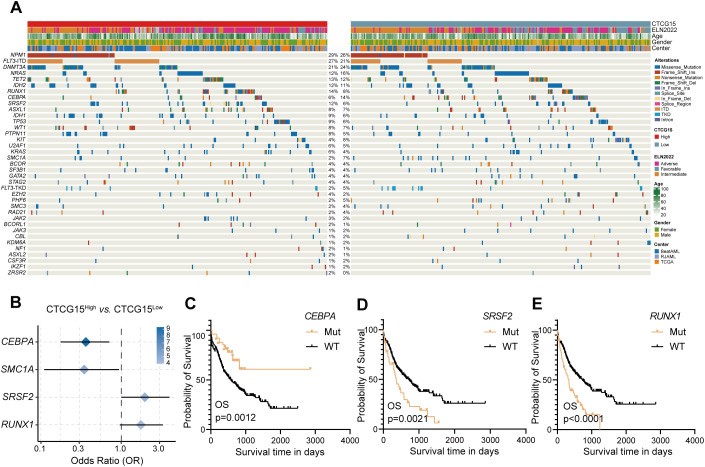
CTCG15 identified distinct genetic abnormalities associated with AML prognosis. **(A)** Somatic mutations and clinical information are presented in a heatmap featuring CTCG15^High^ and CTCG15^Low^ patient groups. **(B)** The forest plot displays gene mutations with significantly different frequencies between CTCG15^High^ and CTCG15^Low^ groups. Kaplan–Meier estimates OS based on the status of *CEBPA* **(C)**, *SRSF2* **(D)** and *RUNX1* **(E)**.

For a more thorough understanding of the mutational landscape linked to the CTCG15 score, we investigated the recurrently mutated somatic driver genes within the combined dataset comprising the BeatAML, RJAML and TCGA cohorts (**Figure 5A**). Four molecular markers showed significant frequency variations between the CTCG15^High^ group and CTCG15^Low^ group (**Figure 5B**). The CTCG15^High^ group exhibited higher frequencies of *SRSF2* and *RUNX1* mutations, which are also markers of the ELN2022 (8) Adverse Risk group. Conversely, patients with low CTCG15 score more commonly presented mutations in the *CEBPA* (including *CEBPA* bZIP mutation and other types) and *SMC1A* genes. In ELN2022 risk classification, bZIP in-frame mutated *CEBPA* is presented in the Favorable Risk group. Notably, the presence of the *CEBPA* mutation indicates a relatively favorable clinical outcome, while *SRSF2* mutation and *RUNX1* mutation an adverse outcome, as demonstrated by univariate analysis (**Figures 5C–E**). In a combined cohort included TCGA cohort, BeatAML cohort and RJAML cohort, a multivariate CPH regression analysis revealed that the CTCG15 score was independent of well-known clinical parameters, ELN2022 classification and the above mentioned three genetic abnormalities (**Supplementary Table S5**).

### The CTCG15 can serve as a valuable complement to the ELN2022 risk classification

**Figure 6:**
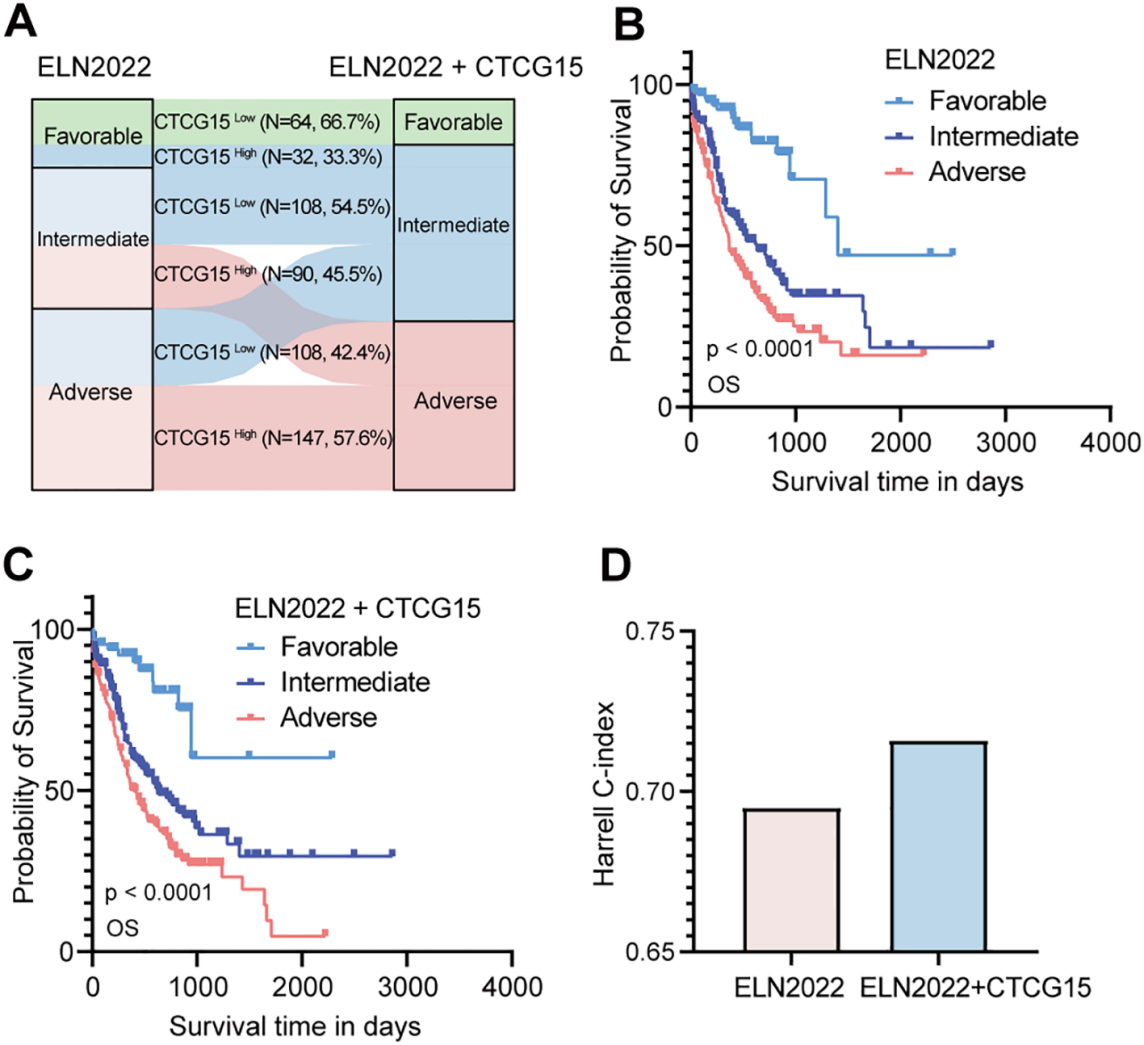
Refine the 2022 European LeukemiaNet (ELN2022) classification by integrating CTCG15. **(A)** Patients in different risk status are recategorized from the original ELN2022 schema categories (Favorable, Intermediate, and Adverse) to the ELN2022 integrated with CTCG15. **(B, C)** Kaplan–Meier estimates of OS based on the risk categories of AML patients in the ELN2022 **(B)** and ELN2022+CTCG15 **(C)** within the combined cohort (RJAML, TCGA-LAML and BeatAML). **(D)** The Harrell C-index assesses performance of risk classification of ELN2022 alone and the ELN2022 integrated CTCG15.

The 2022 ELN risk classification, which is predicated on genetic abnormalities, is widely accepted for the risk assessment of pediatric and adult patients with AML (8). Given the strong association between CTCG15 score and the prognosis of AML patients, we integrated the CTCG15 signature into the ELN2022 scheme with the intention to refine the AML patients risk classification from the immunological perspective. Among all patients categorized within the ELN2022 framework, the incorporation of the CTCG15 score led to a reclassification for approximately half of the patients. Within the ELN2022-Favorable group, 33.3% of patients were reassigned to the Intermediate category of the ELN2022+CTCG15. In the ELN2022-Intermediate group, 45.5% of patients were shifted to the Adverse category of the ELN2022+CTCG15. Furthermore, in the ELN2022-Adverse group, 42.4% of patients were reclassified to the Intermediate category of the ELN2022+CTCG15 (**Figure 6A**). The restructured risk stratification method improves the classification of patients with AML into distinct risk groups from the perspective of immunological factors (**Figures 6B, C**). The revised risk scheme exhibits an elevated Harrell C-index in the combined cohorts, signifying an enhancement in the model’s predictive accuracy and reliability for assessing patient prognosis (**Figure 6D**). This refinement has the potential to make an effective tool for tailoring individualized treatment plans and predicting patient outcomes.

## Discussion

To our understanding, leukemogenesis is driven by the genomic and epigenetic abnormalities. However, the tumor microenvironment, especially the CD8^+^ T in the BM, plays a critical role in adaptive immune reactions, and further affect the efficacy of immunotherapy (3, 33, 34). In this investigation, we tried to elucidate the impacts of BM CD8^+^ T cells in AML.

To quantify the abundance of CD8 TILs in AML, we chose CIBERSORTx (18) among various computational methods (35). Several studies have reported its accuracy in calculating the levels of immune cells in AML (21, 31). Zeng et al. reported in their study that 73% of the cell type estimates generated by CIBERSORTx with S-mode batch correction did not deviate by more than 5% from the scRNA-seq results (31). To validate the accuracy of the CIBERSORTx algorithm, we firstly investigated the discrepancy between the deconvoluted cell type abundance and true cell type abundance from scRNA-seq data. A significant positive association between predicted and observed relative abundance is demonstrated by the Pearson correlation. We further employed two supplementary algorithms (36, 37) to certify the stratification effectiveness. The results aligned coherently with the outcomes produced by the CIBERSORTx evaluation. At the protein level, we adopted the flow cytometry technique (21) to corroborate the proportions of CD8^+^ T cells in BM across both CD8^+^ T^High^ and CD8^+^ T^Low^ groups and the results were consistent with our findings at RNA-level. Concurrently, we employed Pearson correlation to validate their association. The results above strongly supported the reliability of CIBERSORTx as a computational tool to estimate CD8^+^ T cell abundance in bulk RNA-seq.

After calculating the abundance of CD8 TILs using CIBERSORTx in the RJAML cohort, we identified DEGs associated with CD8^+^ T cells for the subsequent analysis. A total of 2925 DEGs that showed significant enrichment in immune activation-related responses and pathways were identified by GO and KEGG enrichment analysis. Using univariate CPH regression and LASSO algorithms, we derived an optimal 15-gene signature (the CTCG15 score) based on CD8 TILs-associated genes. The CTCG15 score was validated using RNA-seq and microarray data from six datasets in total. These datasets encompass a diverse range of ethnicities, including Asians, Africans, and Caucasians, with RJAML dataset specifically being sourced from Ruijin Hospital in Shanghai, China. With multiple validation across different datasets and various populations, the CTCG15 score has demonstrated excellent predictive ability and can be applied to extensive cohorts.

CTCG15 consists of fifteen CD8^+^ T cell marker genes, including *PLIN2*, *MSLN*, *MYH10*, *RXFP1*, *OLFML2A*, *ST6GALNAC4*, *WASIR2*, *MRC1*, *CA3*, *SHANK3*, *C8orf88*, *IL2RA*, *ITGA2B*, *PRUNE2* and *SEMA4F*. Most of these genes have shown associations with the prognosis of AML or the activity of CD8^+^ T cells, which is in accordance with our results. Among the 15 genes used to construct the prognostic model, higher expression of PLIN2, MSLN and MYH10 are associated with favorable prognosis. Research has revealed that the upregulation of lipid droplets associated genes (*PLIN2*) is associated with the enhanced cytotoxic T lymphocytes activity (38). Mesothelin (MSLN) is a new cell surface indicator of the disease and a prospective therapeutic target for AML (39). Recent studies have shown that mesothelin is a key therapeutic target in pediatric AML, and two MSLN/CD3-targeting bispecific antibodies have achieved complete remission in mouse models (40). Genes negatively correlated with CTCG15 are associated with T cell activation.

As for the remaining 12 genes whose expression is negatively associated with prognostic, IL2RA and MRC1 have been extensively studied and are closely related to immune cells. In AML, IL2RA’s overexpression correlates with poor treatment response and adverse outcomes (41, 42), and it ranks as a crucial gene in survival prediction analyses (43). Recently, studies have revealed CD206, encoded by the *MRC1* gene, is an independent adverse prognostic indicator for AML patients (44). Among the other 10 genes positively correlated with CTCG15, each exhibits a profound association with diverse facets of neoplastic initiation and subsequent progression. *SEMA4F* (45) and *C8orf88* (46) are significant contributor to prostate cancer progression. Research has indicated that carbonic anhydrases(CA) play an indispensable role in ensuring leukemic cell viability within an oxygen-deprived environment (47). *ST6GALNAC4* (48), *OLFML2A* (49) and *RXFP1* (50) exhibit a notable association with the initiation and progression of various neoplastic entities. The influential role of most genes that positively correlated with CTCG15 in tumorigenesis and tumor development may contribute to the shorter OS observed in CTCG15^High^.

The goal of establishing an AML prognostic signature is to enhance the effectiveness of both diagnosis and treatment, as well as to provide a more accurate prognosis. Somatic mutations and chromosomal abnormalities drive the onset and development of AML (51, 52). Therefore, we investigated the gene abnormalities related to AML between CTCG15^High^ and CTCG15^Low^. We found four mutations with significantly different frequencies between the CTCG15^High^ and CTCG15^Low^ subgroups. As mentioned, the CTCG15^High^ group is associated with poor prognosis in AML across multiple validation datasets. The occurrence of *SRSF2* and *RUNX1* alterations is higher in this group, aligning with the conclusions of 2022 ELN risk classification (8) and other studies (53–55). Conversely, the CTCG15^Low^ group exhibits a higher incidence of *CEBPA* and *SMC1A* alterations. We have demonstrated that patients with *CEBPA* mutation (including *CEBPA* bZIP mutation and other types) have a prolonged survival. Studies have confirmed that mutations in the bZIP domain of *CEBPA* are associated with a favorable prognosis for patients (8, 56). In the TCGA and BeatAML datasets, the mutation details for *CEBPA* were not provided. However, bZIP mutations account for 90.9% of all *CEBPA* mutations in RJAML. In results, we observed that patients with *CEBPA* mutations have a better prognosis than those without mutations, which may be due to the high proportion of bZIP mutations in the combined cohort. However, the situation is somewhat complicated for *SMC1A*. Structural maintenance of chromosomes protein 1A (SMC1A) is a core unit of the cohesin complex regulating chromosome segregation during meiosis and mitosis (57), which has not been reported with association of prognosis in AML (58, 59). Our results show that alterations in *SMC1A* are more prevalent in the CTCG15^Low^ group, and the underlying mechanism of this observation needs to be further investigated.

To further validate the clinical applicability of CTCG15, we integrated it with the ELN2022 risk classification, leading to more effective patient stratification and increased precision in prognostic accuracy. Several prognostic scores such as LSC17 (60), GENE4 (61), and AFG16 (62) have estimated the outcomes for AML patients recently. Compared to these, our model aims to improve ELN2022’s stratification and prediction ability by incorporating insights from the CD8 TILs in tumor microenvironment. Patients who were reclassified in the three groups were closely associated with CD8 TIL. It has been reported that the function of CD8^+^ T cells is associated to the gene expression in leukemia cells (14, 15). In patients with favorable-risk AML, the proliferation and stemness of leukemic stem and progenitor cells are driven by a limited number of intrinsic molecular abnormalities. Additionally, bone marrow-infiltrating CD8 T cells play a key role in regulating these leukemic stem and progenitor cells (14). However, more aggressive AML is propagated mainly by cell-intrinsic mechanisms and develops independent of immune cells (14). These factors may contribute to the reallocations in ELN2022+CTCG15. Multiple studies have demonstrated that the extent of CD8^+^ T cell infiltration in tumors is closely correlated with patients prognosis and the efficacy of chemotherapy (63–65). These findings coincide with our discovery that ELN2022+CTCG15 improved predictive performance.

To conclude, our study validated the accuracy of CIBERSORTx to estimate the CD8 TILs abundance from the bulk RNA-seq data. We defined fifteen DEGs associated with CD8 TIL abundance and constructed the CTCG15 prognostic model. The CTCG15 score can predict the survival of AML patients, capture gene abnormalities in AML, and significantly enhance the predictive precision of ELN2022. Our research highlights the capability of CTCG15 score to serve as a valuable tool to refine risk stratification in AML and to indicate patient selection for the potential immunotherapy.

## Data Availability

The datasets presented in this study can be found in online repositories. The names of the repository/repositories and accession number(s) can be found below: GEO database under accession number GSE201492.
